# The emergence of T790M mutation in *EGFR*-mutant lung adenocarcinoma patients having a history of acquired resistance to EGFR-TKI: focus on rebiopsy timing and long-term existence of T790M

**DOI:** 10.18632/oncotarget.10351

**Published:** 2016-06-30

**Authors:** Jeng-Sen Tseng, Kang-Yi Su, Tsung-Ying Yang, Kun-Chieh Chen, Kuo-Hsuan Hsu, Hsuan-Yu Chen, Chi-Ren Tsai, Sung-Liang Yu, Gee-Chen Chang

**Affiliations:** ^1^ Division of Chest Medicine, Department of Internal Medicine, Taichung Veterans General Hospital, Taichung, Taiwan; ^2^ Faculty of Medicine, School of Medicine, National Yang-Ming University, Taipei, Taiwan; ^3^ Department of Clinical Laboratory Sciences and Medical Biotechnology, College of Medicine, National Taiwan University, Taipei, Taiwan; ^4^ Department of Laboratory Medicine, National Taiwan University Hospital, Taipei, Taiwan; ^5^ Institute of Biomedical Sciences, National Chung Hsing University, Taichung, Taiwan; ^6^ Division of Critical Care and Respiratory Therapy, Department of Internal Medicine, Taichung Veterans General Hospital, Taichung, Taiwan; ^7^ Institute of Statistical Science, Academia Sinica, Taipei, Taiwan; ^8^ College of Medicine, National Taiwan University, Taipei, Taiwan; ^9^ College of Life Science, National Taiwan University, Taipei, Taiwan; ^10^ Department of Pediatrics, Taichung Veterans General Hospital, Taichung, Taiwan; ^11^ Institute of Molecular Biology, National Chung-Hsing University, Taichung, Taiwan; ^12^ Center of Genomic Medicine, National Taiwan University College of Medicine, Taipei, Taiwan; ^13^ Department of Pathology and Graduate Institute of Pathology, College of Medicine, National Taiwan University, Taipei, Taiwan; ^14^ Center for Optoelectronic Biomedicine, College of Medicine, National Taiwan University, Taipei, Taiwan; ^15^ Comprehensive Cancer Center, Taichung Veterans General Hospital, Taichung, Taiwan

**Keywords:** T790M, epidermal growth factor receptor (EGFR), lung adenocarcinoma, acquired resistance, rebiopsy

## Abstract

Different growth kinetics occurring between the sensitive and T790M-containing cells may result in the repopulation of tumor cells over time. Little information has yet been uncovered on whether rebiopsy timing influences the T790M detection rate. We enrolled a total of 98 *epidermal growth factor receptor (EGFR)*-mutant lung adenocarcinoma patients, who had a history of acquired resistance to EGFR-tyrosine kinase inhibitor (TKI) and available rebiopsy tumor specimens for reassessment of *EGFR* mutations. Rebiopsy was performed at the time of first EGFR-TKI progression in 54 patients (55.1%); for the other 44 patients (44.9%), rebiopsy was done with an interval from first EGFR-TKI progression (median 470.5 days, range 46-1742 days). Our results indicated that rebiopsy timing did not influence the detection rate of T790M and that the mutation could be identified in patients with a long EGFR-TKI-free interval. For patients without suitable lesions for rebiopsy at the time of EGFR-TKI progression, an attempt to rebiopsy should be considered during the subsequent treatment courses.

## INTRODUCTION

Lung cancer is the leading cause of cancer-related death worldwide [1]. Over a recent decade, its treatment has been focused on the personalization of lung cancer therapy, which depends on patient-specific characteristics, including the histological and molecular typing [2, 3]. *Epidermal growth factor receptor (EGFR)* mutation is the most common oncogenic driver in East Asians with lung cancer [4, 5]. As EGFR-tyrosine kinase inhibitor (TKI) can provide a better efficacy and quality of life to the patients, it has emerged as one of the most important therapies among non-small cell lung cancer (NSCLC) patients harboring activating *EGFR* mutations [6–8].

Although most of the NSCLC patients who have sensitive *EGFR*-mutations initially experience a good response to EGFR-TKI, acquired resistance inevitably occurs which leads to a progression of the disease [9, 10]. Various mechanisms have been identified to be associated with acquired resistance, where the most common is a secondary *EGFR* mutation that involves a substitution of threonine to methionine at codon 790 (T790M), which accounts for 50–60% of the acquired resistance mechanisms [11, 12]. Currently, rebiopsy is recommended to confirm the emergence of T790M mutation because of its important role in the prediction of subsequent outcome and the efficacy of third generation EGFR-TKIs [13, 14].

Most previous studies analyzed the T790M mutation at the time that resistance was acquired [11, 12, 15]. Since the T790M-containing resistant cells are believed to grow more slowly than the sensitive cells, repopulation of tumor cells with different growth kinetics, along with restoration of EGFR-TKI sensitivity may occur after EGFR-TKI withdraw [16]; hence, many studies reported that the presence of T790M mutation is associated with a more favorable outcome among patients with acquired resistance to EGFR-TKI [13, 17, 18]. It is still unknown whether rebiopsy timing and the phenomenon of tumor cells repopulation will influence the detection rate of T790M. We conducted this study in order to evaluate the association between patients' characteristics and the T790M mutation rate, which focused on the impact of rebiopsy timing.

## RESULTS

### Patient demographics

Between February 2014 and February 2016, a total of 98 lung adenocarcinoma patients who had experienced acquired resistance to EGFR-TKI and undergone tumor rebiopsy were enrolled for analysis. The baseline characteristics, including the condition of EGFR-TKI treatment and rebiopsy, are shown in Table 1. The median age of the patients was 57.5 years. Sixty-one patients (62.2%) were female and 74 patients (75.5%) were non-smokers. Exon 19 deletions (19Del) and exon 21 L858R were the most common mutation types. Another 4 patients (4.1%) harbored other mutation types and 2 of those were complex mutations involving 19Del or L858R. Most patients (83.7%) received EGFR-TKI as the first line of therapy and 29 patients (29.6%) received more than 1 EGFR-TKIs before rebiopsy.

**Table 1:** Patients' demographic data and characteristics

Characteristics	*n* = 98
Age, median (range) (yrs)	57.5 (30–83)
Gender, *n* (%)	
Male	37 (37.8)
Female	61 (62.2)
Smoking status, *n* (%)	
Non-smokers	74 (75.5)
Former and current smokers	24 (24.5)
Baseline *EGFR* mutations, *n* (%)	
Exon 19 deletions	58 (59.2)
Exon 21 L858R	36 (36.7)
Others^a^	4 (4.1)
First EGFR-TKI regimen, *n* (%)^b^	
Gefitinib	55 (56.1)
Erlotinib	39 (39.8)
Afatinib	4 (4.1)
Initial EGFR-TKI treatment, *n* (%)^b^	
First line	82 (83.7)
Second line or later	16 (16.3)
Total EGFR-TKI(s) treatment, *n* (%)	
1	69 (70.4)
2 or 3	29 (29.6)
Rebiopsy timing (1), *n*(%)^b^	
At first EGFR-TKI PD	54 (55.1)
With interval from first EGFR-TKI PD	44 (44.9)
Rebiopsy timing (2), *n*(%)	
With EGFR-TKI treatment at rebiopsy	78 (79.6)
Without EGFR-TKI treatment at rebiopsy	20 (20.4)
Rebiopsy location (1),*n* (%)	
Primary tumor	29 (29.6)
Metastatic site(s)	69 (70.4)
Rebiopsy location (2),*n* (%)	
Within thorax	70 (71.4)
Out of thorax	28 (28.6)
Rebiopsy specimens, *n*(%)	
Histology	57 (58.2)
Cytology	41 (41.8)
- Fluid	− 35 (85.4)
- Not-fluid	− 6 (14.6)

EGFR, epidermal growth factor receptor; TKI, tyrosine kinase inhibitor; PD, disease progression.

a Include complex mutations involving 19Del or L858R.

b Denote the 1st treated EGFR-TKI; 29 (29.6%) patients have received more than 1 EGFR-TKIs treatment before rebiopsy.

Rebiopsy was performed at the time of first EGFR-TKI progression in 54 patients (55.1%), while for the other patients (44.9%), rebiopsy was performed with an interval from first EGFR-TKI progression (median 470.5 days, range 46-1742 days). Seventy-eight patients (79.6%) were still receiving EGFR-TKI at the time of rebiopsy and of those, 58 patients were under the first EGFR-TKI treatment (including 4 patients kept EGFR-TKI beyond progression with add-on chemotherapy) and 20 patients were with EGFR-TKI retreatment. Rebiopsy was performed via metastatic sites in 69 patients (70.4%). The location of rebiopsy involved lesions within the thorax, which meant that the rebiopsy results came from the primary lung tumor or the M1a metastatic lesions, in 70 patients (71.4%). The types of rebiopsy specimens were histological in 57 patients (58.2%) and cytological in 41 patients (41.8%).

### Initial and rebiopsy EGFR mutation status

**Figure 1:**
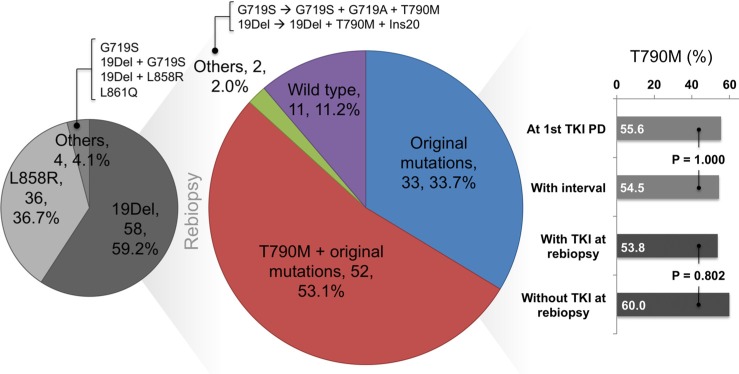
Rebiopsy *epidermal growth factor receptor* (*EGFR*) mutation status and the association with rebiopsy timing

The initial and rebiopsy *EGFR* mutation status are summarized in Table 1 and Figure 1. 19Del and L858R accounted for 59.2% and 36.7% of baseline *EGFR* mutations, respectively. Other mutations included 1 G719S, 1 L861Q, and 2 complex mutations (19Del + G719S and 19Del + L858R). In the rebiopsy specimens, 54 patients (55.1%) were found to be positive for T790M mutation. Of which, all 54 patients contained original mutations with an acquisition of T790M, while 2 of them obtained one more other mutations (1 with add-on G719A and 1 with add-on Ins20). Thirty-three patients (33.7%) only harbored original mutations and 11 patients (11.2%) were *EGFR*-wild type.

### T790M mutation and EGFR-TKI treatment condition

Univariate analyses with regards to the patients' characteristics along with the condition of EGFR-TKI treatment are summarized in Table 2. Neither patients' characteristics nor the efficacy of EGFR-TKI treatment were significantly associated with the emergence of T790M mutation. Of note, patients with L858R mutation and those treated with erlotinib initially displayed a trend towards a lower T790M mutation rate. However, their P values were not significant (*P* = 0.118 and 0.164, respectively). In the present study, 29 (29.6%) patients have received more than 1 EGFR-TKI treatment before rebiopsy. In the subgroup analysis on the remaining 69 patients, who have only received 1 EGFR-TKI, there were still no signs of a significant association between the T790M mutation rate and EGFR-TKI regimens (*P* = 0.203).

**Table 2:** Univariate analysis of the association of rebiopsy T790M mutation with patients' characteristics and the condition of EGFR-TKI treatment

Characteristics	*n*	T790M mutation (%)	*P* value^a^
Age (yr)			0.839
< 60	53	56.6	
≥ 60	45	53.3	
Gender			1.000
Male	37	54.1	
Female	61	55.7	
Smoking status			0.159
Nonsmokers	74	59.5	
Current/former smokers	24	41.7	
Baseline *EGFR* mutations			0.118
Exon 19 deletions	58	62.1	
Exon 21 L858R	36	41.7	
Others^b^	4	75.0	
Initial EGFR-TKI treatment^c^			0.280
First line	82	52.4	
Second line or later	16	68.8	
First EGFR-TKI regimen^c^			0.164
Gefitinib	55	61.8	
Erlotinib	39	43.6	
Afatinib	4	75.0	
First EGFR-TKI response^c^,^d^			0.767
PR	81	54.3	
Durable SD	13	61.5	
First EGFR-TKI PFS^c^			0.223
≥ 10 months	52	61.5	
< 10 months	46	47.8	
Total EGFR-TKI(s) treatment			0.824
1	69	56.5	
2 or 3	29	51.7	

EGFR, epidermal growth factor receptor; TKI, tyrosine kinase inhibitor; PR, partial response; SD, stable disease; PFS, progression-free survival.

a By Fisher's exact test.

b Include complex mutations involving 19Del or L858R.

c Denote the 1st treated EGFR-TKI; 29 (29.6%) patients have received more than 1 EGFR-TKIs treatment before rebiopsy.

d Exclude 4 patients with only evaluable disease.

### T790M mutation and rebiopsy condition

Univariate analyses with regards to the rebiopsy condition are summarized in Table 3. Our results suggested that the rebiopsy timing, location, and types of specimens did not significantly influence the T790M mutation rate. In the case of rebiopsy timing, we evaluated the interval between rebiopsy and progression to initial EGFR-TKI, the best response to treatments after initial EGFR-TKI progression, and whether or not patients continued receiving EGFR-TKI treatment at the time of rebiopsy. We discovered that none of them were significantly associated with the T790M detection rate (*P* = 1.000, 1.000, and 0.802, respectively).

**Table 3:** Univariate analysis of the association of rebiopsy T790M mutation with rebiopsy condition

Characteristics	*n*	T790M mutation (%)	*P* value^a^
Rebiopsy timing (1)^b^			1.000
At first EGFR-TKI progression	54	55.6	
With interval from first EGFR-TKI PD	44	54.5	1.000^c^
- Post-PD best response PR	− 11	− 54.5	
- Post-PD best response SD/PD	− 33	− 54.5	
Rebiopsy timing (2)			0.802
With EGFR-TKI treatment at rebiopsy	78	53.8	
Without EGFR-TKI treatment at rebiopsy	20	60.0	
Rebiopsy location (1)			0.266
Primary tumor	29	44.8	
Metastatic site(s)	69	59.4	
Rebiopsy location (2)			0.509
Within thorax	70	52.9	
Out of thorax	28	60.7	
Rebiopsy specimens			0.681
Histology	57	52.6	
Cytology	41	58.5	0.373^d^
- Fluid	− 35	− 54.3	
- Non-fluid	− 6	− 83.3	

EGFR-TKI, epidermal growth factor receptor tyrosine kinase inhibitor; PD, disease progression; PR, partial response; SD, stable disease.

a By Fisher's exact test.

b Denote the association with 1st treated EGFR-TKI; 29 (29.6%) patients have received more than 1 EGFR-TKIs treatment before rebiopsy.

c Compare the yield rate of rebiopsy T790M mutation among subgroup with interval from EGFR-TKI PD, which were stratified according to the best response to treatments between EGFR-TKI progression and rebiopsy.

d Compare the yield rate of rebiopsy T790M mutation among cytology subgroup.

### Long-term existence of T790M after progression to initial EGFR-TKI treatment

**Figure 2:**
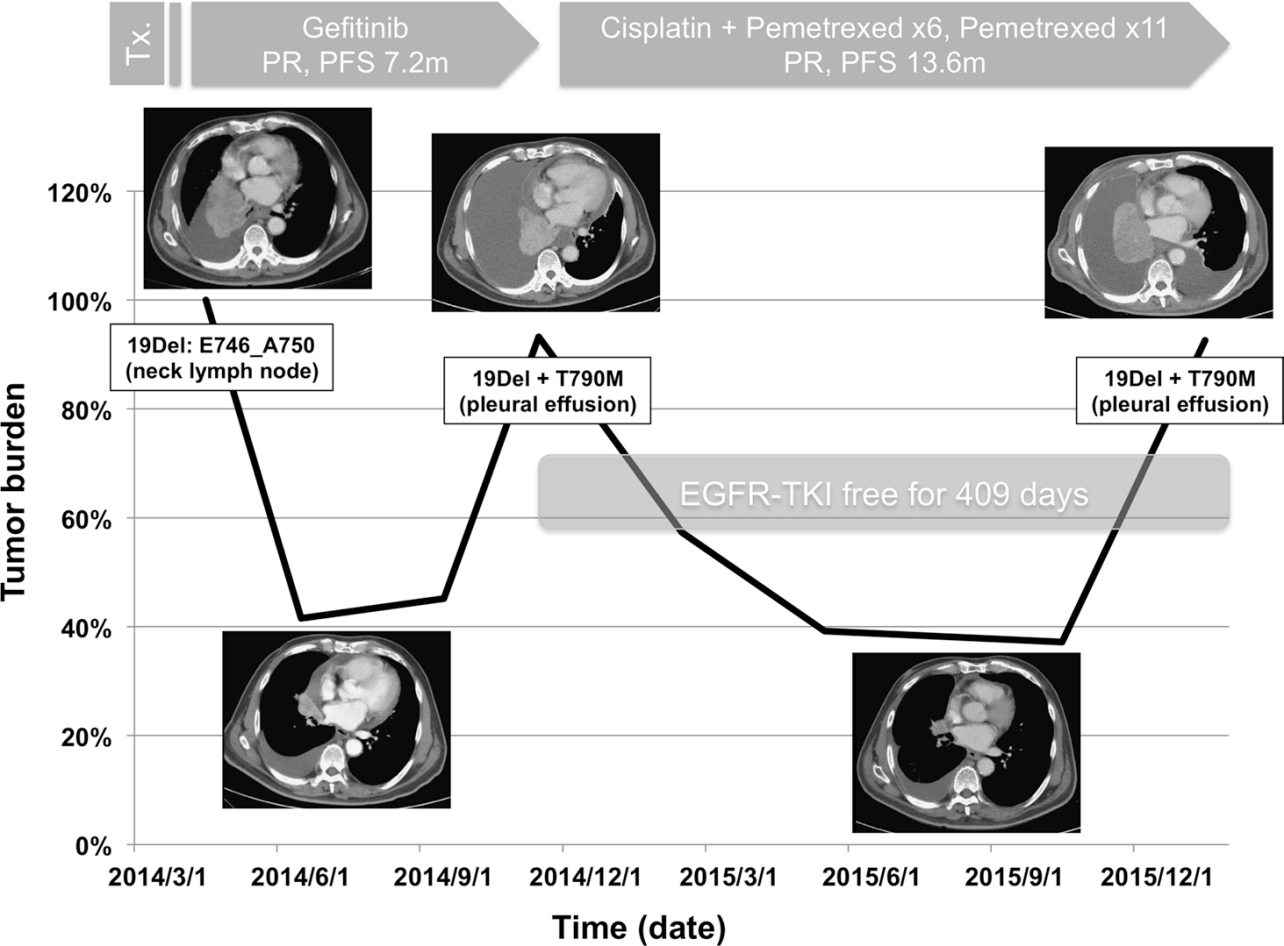
Case presentation demonstrated the long-term existence of T790M after an objective response to chemotherapy and a long EGFR-TKI-free period (PR, partial response; PFS, progression-free survival)

Figure 2 demonstrates the treatment course and serial *EGFR* mutation status of a 66 year-old man experiencing advanced lung adenocarcinoma. The patient achieved a partial response with gefitinib treatment and T790M was “acquired” at the time of disease progression. Second line therapy with chemotherapy also achieved a partial response and the progression-free survival (PFS) was 13.6 months. At the time of disease progression to chemotherapy, a recheck of *EGFR* mutation status over right pleural effusion still yielded T790M mutation, despite a long EGFR-TKI-free period (409 days) and an experience of objective response to chemotherapy. This phenomenon suggested that T790M might exist in the long-term even for patients experiencing an interval from progression to EGFR-TKI and who lack the continuing selection pressure of EGFR-TKI treatment. In the present study, a total of 20 patients were not under EGFR-TKI treatment at the time of rebiopsy and their median interval from the progression to initial EGFR-TKI treatment was 325 days (range 74–818 days). The best responses to treatment during this period were 4 with partial response, 14 with stable disease, and 2 with progressive disease. The T790M detection rate among this subgroup was 60.0%, which was similar to that of the overall population. The median EGFR-TKI free interval was 221 days (range 38–657 days). There were 9, 3, and 1 patient(s) having detectable T790M after an EGFR-TKI free interval more than 180, 360, and 540 days (longest as 657 days), respectively. In multivariate analysis (Table 4), rebiopsy timing did not influence the detection rate of T790M mutation, either in the overall population or in the subgroup patients who received only 1 EGFR-TKI (*P* = 0.612 and 0.862, respectively). Similarly, the area under curve (AUC) in ROC curve analysis regarding the association between the length of rebiopsy interval and T790M detection rate (Supplementary Figure 1) was only 0.453 and 0.425, respectively. All these results suggested that there is no significant association between the rebiopsy timing and the detection rate of T790M mutation.

**Table 4:** Multivariate analysis of the rebiopsy timing and T790M mutation

Rebiopsy timing	Odds ratio	95% CI	*P* value^a^
**All patients (*n* = 98)** *Univariate analysis*			
At first EGFR-TKI PD vs. with interval	1.04	0.47–2.32	0.920
*Multivariate analysis*			
At first EGFR-TKI PD vs. with interval	1.23	0.50–3.01	0.651^b^
	1.58	0.27–9.09	0.612^c^
**Patients received only 1 EGFR-TKI (*n*= 69)**			
*Univariate analysis*			
At EGFR-TKI PD vs. with interval	1.20	0.38–3.84	0.759
*Multivariate analysis*			
At EGFR-TKI PD vs. with interval	1.21	0.32–4.56	0.774^d^
	1.13	0.29–4.41	0.862^e^

PD, disease progression.

a By logistic regression analysis model.

b Adjusted by smoking status, baseline *EGFR* mutations, line of first EGFR-TKI treatment, EGFR-TKI PFS, and rebiopsy location.

c Adjusted by all factors regarding with patients' characteristics, rebiopsy condition, and EGFR-TKI treatment condition (EGFR-TKI regimen was not included because 29.6% patients have been treated with 2 or more EGFR-TKIs).

d Adjusted by smoking status, baseline *EGFR* mutations, EGFR-TKI regimen, line of first EGFR-TKI treatment, EGFR-TKI PFS, and rebiopsy location.

e Adjusted by all factors regarding with patients' characteristics, rebiopsy condition, and EGFR-TKI treatment condition.

### Dynamic T790M status in patients with more than 1 rebiopsy specimens

In our study, there were 24 patients (24.5%) with more than 1 rebiopsy specimens after progression to first EGFR-TKI. The interval and treatment between rebiopsies, type of specimens, and the dynamic T790M status are summarized in Supplementary Table 1. Rebiopsies were performed through the same location in 11 patients (45.8%) and most of them were pleural effusions. Of them, 18 patients (75.0%) had identical T790M status between rebiopsies. Among the 6 patients with discordant T790M results, T790M became undetectable in 4 patients and became detectable in 2 patients. Of note, 2 patients (patient #1 and #10) had rebiopsies before and after AZD9291 treatment. Both of them harbored 19Del+T790M before treatment. One patient still had T790M mutation after AZD9291 treatment but another patient did not have detectable T790M at repeated rebiopsy. Locations of rebiopsy before and after AZD9291 treatment were different in both patients.

## DISCUSSION

As EGFR-TKI resistance in patients with *EGFR*-mutant NSCLC is one type of a “therapy-dependent” clinical condition, acquired resistance is defined as being progressive “while” receiving EGFR-TKI [9, 19]. Therefore, rebiopsy was done at the time of disease progression in most studies [11, 12, 15], and the results revealed that T790M has accounted for 50–60% of the resistance mechanisms. Previous studies have shown the different growth kinetics between EGFR-TKI sensitive and resistant cells, which implies that the T790M-containing resistant cells would grow more slowly. Based on the “tumor cells repopulation” hypothesis, several clinical phenomenons were reported, including the restoration of EGFR-TKI sensitivity after EGFR-TKI withdrawal [15], disease flare after discontinuation of EGFR-TKI [20], and a better outcome for T790M-mutant patients [17]. Since the cancer genome is heterogeneous, can evolve over time, and also interacts with different treatments [21]; it remains unclear whether rebiopsy timing will influence the detection rate of T790M. In the present study, our results provided the evidence that there was no significant association between the rebiopsy timing and the T790M detection rate.

T790M can confer a substantial enhancement of kinase activity when combined with the characteristic *EGFR* activating mutations [22, 23]. In animal models, mice expressing the T790M mutation in type II pneumocytes alone were shown to develop lung adenocarcinoma [24]. Moreover, germline T790M mutation was reported to be associated with an inherited susceptibility to lung adenocarcinoma in a family with multiple members suffering from lung cancer [25]. Recently, the promising efficacy of the third generation EGFR-TKIs also bolstered T790M as an actionable driver [14, 26]. In the present study, we showed that T790M mutation may exist long-term even in patients experiencing a long EGFR-TKI-free interval. All these data suggest that T790M is not only a cause of acquired resistance of EGFR-TKI but also offers significant oncogenic activity.

Little is known about the T790M mutagenesis and it remains doubtful whether T790M previously existed as a minor clone before treatment or evolved during the course of EGFR-TKI treatment. Several studies have independently identified pre-treatment T790M and suggested that it is a poor outcome predictor of EGFR-TKI treatment [27–29]. A study by Hata et al. suggested that T790M could both pre-exist and evolve from the drug-tolerant cells, and that the different mechanisms may result in distinct efficacy of third generation EGFR-TKI treatment [30]. Despite the promising efficacy of third generation EGFR-TKIs in T790M-mutant patients [14, 26], primary resistance did exist, which hinted that T790M is not a homogeneous status and can coexist with other resistance mechanisms [31, 32]. Cancer genomes can vary over time. Although T790M mutation can be detected in various timings, how it interferes with the outcome of third generation EGFR-TKI treatment requires further investigation.

The T790M mutation rate in our cohort was 55.1%, which was similar to that of previous studies [11–13, 17]. By comparison, our cohort was relatively homogeneous because of the pure adenocarcinoma histology and Asian ethnicity. Similarly, in the study by Hata et al., there was also no significant difference in the T790M mutation rate, whether the rebiopsy was performed within 4 months of EGFR-TKI failure or not [13]. Herein, we further disclosed that the T790M detection rate would not be reduced even for patients experiencing an objective response to post-EGFR-TKI therapies, or in patients not continuing selection pressure of EGFR-TKI treatment. For patients without suitable lesions for rebiopsy at the time of EGFR-TKI progression, an attempt to rebiopsy should be considered during the subsequent treatment courses.

In the present study, we also evaluated the role of the patients' demographics and the EGFR-TKI treatment condition in the prediction of T790M mutation and disclosed that all of the patient characteristics, baseline *EGFR* mutation types, EGFR-TKI regimens, EGFR-TKI efficacy, line of EGFR-TKI treatment, and total EGFR-TKI exposures did not affect the T790M detection rate. These results were similar to that of previous studies [13, 17], and suggested that T790M mutation cannot be predicted solely through clinical characteristics. As T790M can serve as an important prognostic factor, while also predicting the efficacy of third generation EGFR-TKI, rebiopsy should be considered for all patients experiencing disease progression to EGFR-TKI treatment.

Our previous study has demonstrated that plasma *EGFR* mutation status can be dynamically assessed and also serve as an independent outcome predictor of EGFR-TKI therapy [33]. Theoretically, this concept can also be applied in both T790M detection and third generation EGFR-TKI therapy. By now, only a small subset of patients are considered feasible for rebiopsy due to the limitation of anatomical locations, risk of rebiopsy, and limited medications that can be directed by rebiopsy [34]; hence, liquid biopsy is a better alternative for obtaining the information on the dynamics of T790M status. Recently, studies by Sueoka-Aragane et al. and Zheng et al. both disclosed that T790M can be detected in plasma before and after clinical progression to EGFR-TKI and act as a poor prognostic factor [35, 36]. However, the detection sensitivity is not at a high enough level and the correlation with the efficacy of third generation EGFR-TKI treatment was lacking. The impact on the outcome of upcoming third generation EGFR-TKI therapy era due to the discrepancy between plasma and rebiopsy tumor T790M status requires further evaluation.

In the present study, the rebiopsy *EGFR* status of 11.2% patients were wild type, which meant that some of the *EGFR* mutations could disappear after EGFR-TKI treatment. This phenomenon could also be observed during the T790M-targeted therapy. In a study by Piotrowska et al., half of T790M-mutant NSCLC patients were T790M-wild type upon progression to rociletinib treatment [37], which underlies the importance of tumor heterogeneity.

The major limitation we have encountered is the retrospective nature of the present study. Rebiopsy could be performed in only 14% of progression patients even within a prospective, observational study [35]. In clinical practice, the results from a small number of patients who are able to receive rebiopsy might not completely reflect the actual conditions of general population. Previous studies have suggested the “wax and wane” nature of T790M mutation [11, 31, 38], and part of our patients also had discordant T790M status between repeated rebiopsies. By now, much remained unknown about the nature of T790M mutagenesis. Because of the potential limitations caused by various sensitivity of detection methods, quality of rebiopsy specimens, and the effect of tumor heterogeneity, further prospective studies are needed to clarify whether the T790M mutation is “gain-and-loss” or “detectable-and-undetectable” during the treatment course. Although our data were collected retrospectively, we tried to ensure the validity of patients' characteristics, along with the correlation between treatment course and T790M mutation status.

In conclusion, our results showed that rebiopsy timing did not influence the detection rate of T790M and that the mutation could be identified in patients with a long EGFR-TKI-free interval. We suggested that T790M could exist long-term after progression to EGFR-TKI therapy, while serving as a significant oncogenic driver.

## MATERIALS AND METHODS

### Patients

We retrospectively analyzed lung cancer patients who were diagnosed and treated at Taichung Veterans General Hospital (TCVGH) between February 2014 and February 2016. To be eligible for participation in the study, patients were required to have pathologically confirmed advanced stage lung adenocarcinoma, known sensitive *EGFR* mutations (including exon 18 G719X, exon 19 deletions, exon 21 L858R, and exon 21 L861Q) in pre-EGFR-TKI treatment tumor specimens, a history of EGFR-TKI therapy, and available rebiopsy tumor specimens for re-assessment of their *EGFR* mutation status. Moreover, all patients had to fall into the clinical definition of acquired resistance to EGFR-TKI [9]. Patients were excluded if they had lung tumor with doubtful origin, other active malignancies, a primary resistance to EGFR-TKI, or T790M mutation in pre-EGFR-TKI treatment tumor specimens.

Clinical data for analysis included patients' age, gender, smoking status, EGFR-TKI and chemotherapy treatment condition, rebiopsy condition, and *EGFR* mutation status. Unidimensional measurement as defined by the Response Evaluation Criteria in Solid Tumors (Version 1.1) was implemented in this study [39].

### EGFR mutation analysis

Tumor specimens were collected and procured for *EGFR* mutation analysis as previously described [28, 40]. The detection method used in this study was Matrix-Assisted-Laser-Desorption-Ionization Time-of-Flight mass spectrometry (MALDI-TOF MS). The detection spectrum of MALDI-TOF MS is summarized in Supplementary Table 2. We performed the testing according to the instructions provided by the MassARRAY^®^ system (Sequenom, San Diego, CA). With respect to the biochemical reaction, PCR was used to amplify the region containing the tyrosine kinase domain of the *EGFR* exons 18, 19, 20, and 21. A single nucleotide extension was then performed by primers and corresponding detection probes to amplify the region containing each target mutation. After SpectroClean Resin clean up, samples were loaded onto the matrix of SpectroCHIP^®^ by Nanodispenser (Matrix) and then analyzed by Bruker Autoflex MALDI-TOF MS. Data was collected and analyzed by Typer4 software (Sequenom, San Diego, CA). All the tests were performed by ISO15189-certified TR6 Pharmacogenomics Lab (PGL), National Research Program for Biopharmaceuticals (NRPB), at the National Center of Excellence for Clinical Trial and Research of NTUH.

### Statistical methods

Univariate analysis by Fisher's exact test was conducted on the frequency of T790M mutation in order to evaluate the effects of clinical factors relating to patients' characteristics, the EGFR-TKI treatment condition, and rebiopsy condition. With regard to rebiopsy timing, patients who had received treatment other than EGFR-TKI between the first EGFR-TKI progression and rebiopsy were defined as “with interval from first EGFR-TKI progression” and *vice versa*. Patients who had continued receiving EGFR-TKIs treatment within 1 month before rebiopsy, were defined as “with EGFR-TKI treatment at rebiopsy” and *vice versa*. A logistic regression model was used to evaluate the impact of rebiopsy timing and the T790M mutation rate. A receiver-operating characteristic (ROC) curve was constructed to evaluate the impact of the interval between the first EGFR-TKI progression and rebiopsy in the predicting of T790M mutation. All statistical tests were done with SPSS 15.0 (SPSS Inc., Chicago, IL, USA). Two-tailed tests and *p* values < 0.05 for significance were used.

## SUPPLEMENTARY MATERIALS


