# Supplementary material for: The emergence of T790M mutation in EGFR-mutant lung adenocarcinoma patients having a history of acquired resistance to EGFR-TKI: focus on rebiopsy timing and long-term existence of T790M
Source: Oncotarget. 2016 Jun 30;7(30):48059–69. doi: 10.18632/oncotarget.10351 (PMC5217000; doi:10.18632/oncotarget.10351)
Supplement: Supplementary file 1 [file oncotarget-07-48059-s001.pdf]

The emergence of T790M mutation in *EGFR*-mutant lung adenocarcinoma patients having a history of acquired resistance to EGFR-TKI: focus on rebiopsy timing and long-term existence of T790M

Supplementary Materials

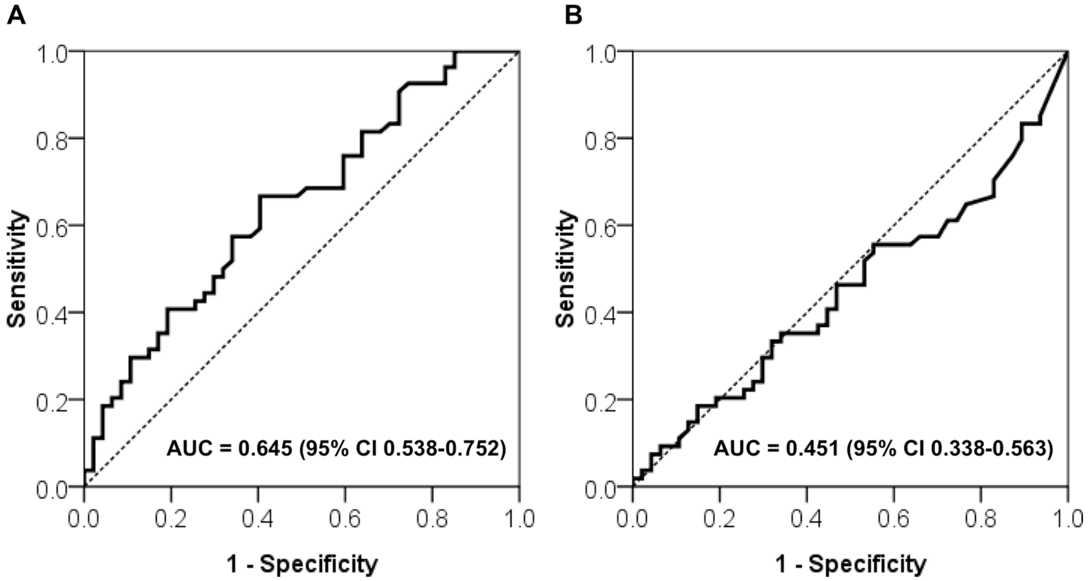

Supplementary Figure S1: ROC curves regarding the association of rebiopsy T790M mutation with the interval between first EGFR-TKI progression and rebiopsy in overall population (**A**) (n = 98) and subgroup patients who received only 1 EGFR-TKI (**B**) (n = 69).

Supplementary Table S1: Rebiopsy T790M status in patients with more than 1 rebiopsy specimens (*n* = 24)

Pt	Age/ Gender	Original mutation	Interval between rebiopsies (days)	Tx. between rebiopsies	Rebiopsy site(s)/T790M status
1	44/F	19Del	295	TKI[*]	Pleura/+ → Lung tumor/+[*]
2	67/F	19Del	213	CT	Lymph node/+ → Lymph node/+
3	59/F	19Del	70	CT	**Effusion/− → Effusion/+**
4	66/M	19Del	435	CT	Effusion/+ → Effusion/+
5	47/F	19Del	254, 208	CT, TKI	**Effusion/− → Effusion/− → Effusion/+[#]**
6	45/F	L858R	169	CT	Lung tumor/− → Effusion/−
7	79/F	L858R	397	CT, TKI	**Effusion/+ → Effusion/−**
8	67/F	L858R	783	CT, TKI	**Lung tumor/+ → Effusion/−**
9	53/M	19Del	383	BEV, CT, TKI	Lung tumor/+ → Lymph node/+
10	47/F	19Del	493	TKI[*]	**Lymph node/+ → Effusion/−[*]**
11	40/F	L858R	245	CT	**Bone/+ → Pleura/−**
12	63/M	19Del	140	TKI	Effusion/+ → Effusion/+
13	55/F	19Del	90	CT	Pleura/+ → Lymph node/+
14	59/F	19Del	296	CT	Effusion/+ → Pleura/+
15	60/M	L858R	225	CT, TKI	Lunt tumor/− → Bone/−
16	45/M	19Del	298	CT, TKI	Lung tumor/− → Lung tumor/−
17	37/F	19Del	169	BEV, CT, TKI	Effusion/− → Breast metastasis/−
18	68/F	L858R	525	CT, TKI	CSF/− → CSF/−
19	83/M	L858R	477	CT, TKI	Lunt tumor/− → CSF/−
20	56/F	L861Q	152	CT, TKI	CSF/− → Effusion/−
21	72/F	L858R	574	CT, TKI	Lymph node/− → Effusion/−
22	81/F	19Del	134, 101	CT, TKI	Effusion/− → Effusion/− → Effusion/−
23	56/M	19Del	284	CT	Effusion/− → Effusion/−
24	46/M	19Del	89	BEV, PD1, TKI	Effusion/− → Effusion/−

BEV, bevacizumab; CT, Chemotherapy(ies); PD1, anti-PD1 immunotherapy; TKI, epidermal growth factor receptor-tyrosine kinase inhibitor(s).

[*]Osimertinib (AZD9291) treatment.

[#]The 3rd pleural effusion specimen was from the contralateral lung.

Marking note: underline, without 1st or 2nd generation EGFR-TKI therapy at rebiopsy; bold, discordant T790M status between rebiopsies.

Supplementary Table S2: *EGFR* mutations detected by MALDI-TOF MS

Exon	Mutation types (nucleic acid sequence)
18	E709A (2126A>C), E709G (2126A>G), E709V (2126A>T), G719A
	(2156G>C), G719C (2155G>T), G719N (2156G>A), G719S (2155G>A)
19	Del E746_A750 (2235_2249del15, 2236_2250del15),
	Del E746_T751 (2236_2253del18),
	Del E746_T751>A (2237_2251del15),
	Del E746-T751>I (2235_2252>AAT),
	Del E746_S752>A (2237_2254del18),
	Del E746_S752>D (2238_2255del18),
	Del E746_S752>V (2237_2255>T),
	Del L747_E749 (2239_2247del9),
	Del L747_A750>P (2238_2248>GC, 2239_2248TTAAGAGAAG>C),
	Del L747_T751 (2239_2253del15, 2240_2254del15),
	Del L747_T751>P (2239_2251>C),
	Del L747_T751>Q (2238_2252>GCA),
	Del L747_T751>S (2240_2251del12),
	Del L747_S752 (2239_2256del18),
	Del L747_P753>Q (2239_2258>CA),
	Del L747_P753>S (2240_2257del18)
20	S768I (2303G>T), T790M (2369C>T)
21	L858Q (2573T>A), L858R (2573T>G), L861Q (2582T>A)

EGFR, epidermal growth factor receptor; MALDI-TOF MS, matrix-assisted laser desorption ionization-time of flight mass spectrometry.
